# Supplementary material for: A fault-intrusion-tolerant system and deadline-aware algorithm for scheduling scientific workflow in the cloud
Source: PeerJ Comput Sci. 2021 Nov 2;7:e747. doi: 10.7717/peerj-cs.747 (PMC8576554; doi:10.7717/peerj-cs.747)
Supplement: Supplemental Information 6 [file peerj-cs-07-747-s006.zip › Myproject1.1/docs/org/workflowsim/utils/ClusteringParameters.html]

ClusteringParameters


JavaScript is disabled on your browser.


- Overview
- Package
- Class
- Use
- Tree
- Deprecated
- Index
- Help

- Prev Class
- Next Class

- Frames
- No Frames

- All Classes

- Summary:
- Nested |
- Field |
- Constr |
- Method

- Detail:
- Field |
- Constr |
- Method


org.workflowsim.utils

## Class ClusteringParameters

- java.lang.Object
- - org.workflowsim.utils.ClusteringParameters

- ---

    

  ```
  public class ClusteringParameters
  extends java.lang.Object
  ```

  ClusteringParameters contains all the parameters used in task clustering

  Since:
  :   WorkflowSim Toolkit 1.0

- - ### Nested Class Summary

    Nested Classes

    | Modifier and Type | Class and Description |
    | --- | --- |
    | `static class` | `ClusteringParameters.ClusteringMethod` Supported Clustering Method, by default it is none |
  - ### Constructor Summary

    Constructors

    Constructor and Description

    `ClusteringParameters(int cNum,
    int cSize,
    ClusteringParameters.ClusteringMethod method,
    java.lang.String code)`

    Initialize a ClusteringParameters
  - ### Method Summary

    Methods

    | Modifier and Type | Method and Description |
    | --- | --- |
    | `ClusteringParameters.ClusteringMethod` | `getClusteringMethod()` Gets the clustering method |
    | `int` | `getClustersNum()` Gets the number of clustered jobs per level |
    | `int` | `getClustersSize()` Gets the size of clustered jobs, which is equal to the number of tasks in a job |
    | `java.lang.String` | `getCode()` Gets the code for balanced clustering Please refer to our balanced clustering paper for details |

    - ### Methods inherited from class java.lang.Object

      `clone, equals, finalize, getClass, hashCode, notify, notifyAll, toString, wait, wait, wait`

- - ### Constructor Detail


    - #### ClusteringParameters

      ```
      public ClusteringParameters(int cNum,
                          int cSize,
                          ClusteringParameters.ClusteringMethod method,
                          java.lang.String code)
      ```

      Initialize a ClusteringParameters

      Parameters:
      :   `cNum,` - clustes.num
      :   `cSize,` - clusters.size
      :   `method,` - clusters.method
      :   `code` - , balanced clustering code (used for research)
  - ### Method Detail


    - #### getCode

      ```
      public java.lang.String getCode()
      ```

      Gets the code for balanced clustering Please refer to our balanced
      clustering paper for details

      Returns:
      :   the code


    - #### getClustersNum

      ```
      public int getClustersNum()
      ```

      Gets the number of clustered jobs per level

      Returns:
      :   clusters.num


    - #### getClustersSize

      ```
      public int getClustersSize()
      ```

      Gets the size of clustered jobs, which is equal to the number of tasks in
      a job

      Returns:
      :   clusters.size


    - #### getClusteringMethod

      ```
      public ClusteringParameters.ClusteringMethod getClusteringMethod()
      ```

      Gets the clustering method

      Returns:
      :   clusters.method


- Overview
- Package
- Class
- Use
- Tree
- Deprecated
- Index
- Help

- Prev Class
- Next Class

- Frames
- No Frames

- All Classes

- Summary:
- Nested |
- Field |
- Constr |
- Method

- Detail:
- Field |
- Constr |
- Method
